# Ecosystem multifunctionality and soil microbial communities in response to ecological restoration in an alpine degraded grassland

**DOI:** 10.3389/fpls.2023.1173962

**Published:** 2023-08-01

**Authors:** Xiangyang Shu, Weijia Liu, Yufu Hu, Longlong Xia, Kunkun Fan, Yanyan Zhang, Yulin Zhang, Wei Zhou

**Affiliations:** ^1^ College of Resources, Sichuan Agricultural University, Chengdu, China; ^2^ Institute of Agricultural Bioenvironment and Energy, Chengdu Academy of Agriculture and Forestry Sciences, Chengdu, China; ^3^ Institute for Meteorology and Climate Research (IMK-IFU), Karlsruhe Institute of Technology, Garmisch-Partenkirchen, Germany; ^4^ State Key Laboratory of Soil and Sustainable Agriculture, Institute of Soil Science, Chinese Academy of Sciences, Nanjing, China; ^5^ Department of Civil Engineering, The University of Hong Kong, Hong Kong SAR, China

**Keywords:** ecological restoration, multifunctionality, biodiversity, microbial stability, alpine grassland

## Abstract

Linkages between microbial communities and multiple ecosystem functions are context-dependent. However, the impacts of different restoration measures on microbial communities and ecosystem functioning remain unclear. Here, a 14-year long-term experiment was conducted using three restoration modes: planting mixed grasses (MG), planting shrub with *Salix cupularis* alone (SA), and planting shrub with *Salix cupularis* plus planting mixed grasses (SG), with an extremely degraded grassland serving as the control (CK). Our objective was to investigate how ecosystem multifunctionality and microbial communities (diversity, composition, and co-occurrence networks) respond to different restoration modes. Our results indicated that most of individual functions (i.e., soil nutrient contents, enzyme activities, and microbial biomass) in the SG treatment were significantly higher than in the CK treatment, and even higher than MG and SA treatments. Compared with the CK treatment, treatments MG, SA, and SG significantly increased the multifunctionality index on average by 0.57, 0.23 and 0.76, respectively. Random forest modeling showed that the alpha-diversity and composition of bacterial communities, rather than fungal communities, drove the ecosystem multifunctionality. Moreover, we found that both the MG and SG treatments significantly improved bacterial network stability, which exhabited stronger correlations with ecosystem multifunctionality compared to fungal network stability. In summary, this study demonstrates that planting shrub and grasses altogether is a promising restoration mode that can enhance ecosystem multifunctionality and improve microbial diversity and stability in the alpine degraded grassland.

## 1 Introduction

Ecological restoration has been widely implemented as an effective strategy to address the loss of biodiversity and ecosystem functions and services (Huang et al., 2019; Strassburg et al., 2020). Previous studies have reported on the influence of ecological restoration on individual ecosystem functions (Yang et al., 2019; Bai et al., 2020), while the benefits of ecological restoration can be more effective when multiple ecosystem functions are comprehensively considered (Tian et al., 2022). Ecosystem multifunctionality (EMF), as a reliable index to summarize the complex and interactive processes of ecosystems, has been increasingly applied to evaluate the influence of human activities and climate change on the multiple functions of ecosystems (Hector & Bagchi, 2007; Garland et al., 2021). Understanding the effects and fundamental mechanisms of ecological restoration on ecosystem multifunctionality can help predict the responses of ecosystems to long-term ecological restoration and therefore facilitate the implementation of large-scale restoration projects.

Soil microbiota constitutes a significant portion of the earth’s biodiversity and plays a crucial role in various ecosystem functions and services, including organic matter decomposition, nutrient cycling, soil aggregate stabilization, and plant productivity (Saleem et al., 2019). The diversity and composition of microbial communities are highly sensitive to anthropogenic disturbances and environmental changes, such as climate change, land use change, and restoration (Farrell et al., 2020; Rillig et al., 2019). Ecological restoration can have substantial effects on soil microbial communities, often leading to consequences for ecosystem functions and services. Furthermore, the response of microbial communities in terms of diversity and composition varies depending on the restoration modes enployed(Bizuti et al., 2022; Lu et al., 2022). Given the significance of soil microbial community diversity and composition in relation to EMF, they should be considered when examining the mechanisms underlying the response of degraded grassland ecosystems to ecological restoration.

Microbial communities exhibit structured interactions, forming complex ecological network through processes such as competition, facilitation, and inhibition (Hirano & Takemoto, 2019). In recent years, the stability of soil microbial network has garnered increasing attention due to its potential implications for ecosystem functions (Karimi et al., 2017). Co-occurrence network analysis, a mathematical modeling approach, has been utilized to understand the stability of microbial communities and how it evolves in response to natural and anthropogenic perturbations (Barberán et al., 2012). Network topological properties, such as modularity and cohesion indices, have been successfully employed to estimate microbial network stability (de Vries et al., 2018). While numerous studies have demonstrated that environmental changes can alter soil microbial network stability and subsequently impact ecosystem functions (Ye et al., 2021; Yuan et al., 2021), only a limited number ofstudies have investigated the response of microbial network stability to ecological restoration and its relationship with EMF.

The alpine grasslands in the northwest Sichuan, located at the eastern margin of the Tibetan Plateau, play a crucial role in protecting water resources and ecological security (Zhou and Peng, 2021). However, due to human disturbances and climate changes, the degradation of grassland in this area increased by 307.7% during the 34 years from 1966 to 2000, with an average annual degradation area increased by 816.0 hm^2^ (Ma et al., 2020). In response to this, the Chinese government had implemented ecological restoration measures in an attempt to reverse the decline in ecosystem functions and services (Gou et al., 2019; Liu et al., 2022). However, there is limited information available regarding the effectiveness of different restoration modes in this unique area. Therefore, this study aims to explore how different restoration modes may influence ecosystem functions and soil microbial communities in the alpine degraded grasslands on the eastern margin of the Tibetan Plateau. Three restoration modes are considered: planting mixed grasses (MG), planting shrub with *Salix cupularis* alone (SA), and planting shrub with *Salix cupularis* in combination with grasses (SG). The specific objectives of this study are: 1) to investigate changes in EMF after a 14-year restoration treatment, 2) to examine the influence of different restoration modes on microbial communities, and (3) to identify the driving mechanism of the relationship between microbial communities and EMF.

## 2 Materials and methods

### 2.1 Study site and experimental design

The study area is located in the Restoration Demonstration region of degraded grassland in Hongyuan County (33° 1’ N and 102° 37’ E), China, at the eastern margin of the Tibetan Plateau (**Supplementary Figure S1**). The average elevation of this region is over 3400 m. The mean annual precipitation in this region is 791.95 mm, with approximately 60–75% occurring from May to September. The mean annual temperature is 1.1°C, and the mean temperatures are -10.3°C and 10.9°C for the coldest and warmest months, respectively. The soil is classified as cambic arenosol (FAO, 2006), which is sandy in texture, loose in structure and low in nutrients. The characteristics of the restoration demonstration region are gently undulating moving, semi-moving, and semi-fixed dunes. The dominant vegetation species in the recovery area are mainly *Salix cupularis*, *Carex peaeclara*, *Kobresia pygmaea*, *Artemisia wellbyi*, and *Heracleum souliei*. Since 2007, different restoration modes had been started to restore degraded grassland. The restoration actions included 1) planting mixed grasses (MG), 2) planting shrub with *Salix cupularis* alone (SA), and 3) planting shrub with *Salix cupularis* plus grasses (SG). After 14 years, these restoration modes have been successfully established in the restored area. Further details on dominant plant communities can be found in **Table 1**.

**Table 1:** Basic information of dominant plant communities under different restoration modes.

Restoration modes	Mean slope (°)	*Salix Cupularis*		Coverage (%)	Dominant species
		Mean height (m)	Mean canopy (m)		
CK	< 5			4.2 ± 1.9	*Cyperus stoloniferus*
MG	< 5			94.3 ± 0.8	*Carex praeclara Nelmes*, *Tibetia himalaica*, *KobresiasetchwanensisHand.–Mazz*, *Potentilla anserina*
SA	< 5	1.52	1.97×1.84	6.6 ± 1.4	*Lancea tibetica*, *Leymus secalinus*, *Salix cupularis*
SG	< 5	1.64	1.98×1.91	81.4 ± 8.9	*Euphrasia regelii subsp. Kangtienensis*, *Salix cupularis*, *Anaphalis lactea*, *Peucedanum praeruptorum*, *Potentilla discolor*, *Elymus nutans*

CK, extremely degraded grassland; MG, planting mixed grasses; SA, planting shrub with Salix cupularis alone (SA); SG, planting shrub with Salix cupularis plus mixed grasses.

In this study, our main purpose was to assess the performance of the three restoration modes after 14 years of successful establishment. From October to April in the study area, freezing and ice-induced erosion may inhibit plant growth and alter the physical, chemical, and biological properties of the habitat, likely leading to habitat homogenization. These changes may not reflect the real effects of restoration modes on ecosystem functions and microbial communities. We collected soil and plant samples in August 2021, because the effects of different restoration modes may be more apparent in the summer season due to enhanced growth of plant and microorganisms, higher temperature, and more stable weather conditions. For each treatment, four 25 m × 25 m plots were selected as the replicates. For the CK and MG plots, four 1 m × 1 m quadrats were randomly performed to survey herbaceous plants. For the SA and SG plots, three 10 m × 10 m subplots were randomly selected to investigate shrub height and canopy, while four 1 m × 1 m quadrats were selected for bare land between two adjacent shrubs to explore herbaceous features in each subplot. At each plot, four 1 m × 1 m quadrats at least 10 m apart from each other were selected. Above-ground plant and litter biomass in the quadrat were determined by cutting the herbs close to the ground and placing them in hard envelopes for ongoing drying at 65°C for 72 h. We randomly sampled 1 kg of soil from the top 0-20 cm in each plot using a soil auger of 5 cm diameter, and then pooled and thoroughly mixed it to produce a composite soil sample. After transporting these samples to the laboratory on ice, one-tenth of each soil sample was stored at -80°C for the soil microbial community analysis. Two-tenths of each soil sample was stored at 4°C for testing soil microbial biomass carbon and nitrogen and enzyme activities. The remaining soil was air-dried and sieved for pH, organic matter, and soil nutrients analysis.

### 2.2 Soil analysis

Soil properties were analyzed as previously described by Carter & Gregorich (2008). Soil pH was determined by a glass electrode with a soil-to-water ratio of 1:2.5 (weight/volume) (Mettler Toledo MP220, Mettler-Toledo, Switzerland). Soil organic matter (SOM) content was analyzed using the K_2_Cr_2_O_7_ oxidation method. Soil total nitrogen (TN) content was measured using a flow injection autoanalyzer (AutoAnalyzer 3, Bran+ Luebbe, Germany). Soil total phosphorus (TP) content was analyzed calorimetrically using the H_2_SO_4_-HClO_4_ method. Microbial biomass carbon (MBC) and nitrogen (MBN) were measured by the chloroform fumigation-extraction method (Brookes et al., 1985). Dissolved organic carbon (DOC) and total nitrogen (DTN) were measured by TOC/TN analyzer (Elementer Analysensysteme, Germany). Additionally, we analyzed the potential activities of five soil extracellular enzymes, including β-glucosidase (BG), β-D-cellubiosidase (CBH), N-acetyl-β-glucosaminidase (NAG), leucine amino peptidase (LAP), and acid phosphatase (ACP) (**Supplementary Table S1**). All enzymes were quantified using commercial enzyme kits following the manufacturer’s protocol (Solarbio Science and Technology Co., Ltd., Beijing, China).

### 2.3 Ecosystem multifunctionality

The multifunctionality includes multiple ecosystem functions such as plant biomass, nutrient cycling, soil fertility and SOM decomposition (Delgado-Baquerizo et al., 2020; Guo et al., 2021). To obtain a quantitative ecosystem multifunctionality index for each plot, we used two independent multifunctionality approaches: (1) the averaging multifunctionality index and (2) the multidimensional multifunctionality index (Delgado-Baquerizo et al., 2020). Before analyses, we averaged the standardized scores (a common scale ranging from 0 to 1) of all individual ecosystem functions (Byrnes et al., 2014). The averaging approach takes the mean value across all standardized functions as a multifunctional index for each plot (Maestre et al., 2012). The multidimensional approach is calculated based on a principal coordinates analysis (PCoA) by using the data of each ecosystem-related function (Manning et al., 2018). The multifunctionality index is calculated by summing up all site-scores of the PCoA after weighting the axes by their eigenvalues. A notable benefit of the multidimensional approach is the avoidance of potential collinearity issues among multiple measured functions (Chen et al., 2022). Here both the averaging and multidimensional indices of ecosystem multifunctionality were highly correlated (r = 0.98, *P* < 0.001). Thus, we present results using the multidimensional calculation of ecosystem multifunctionality.

### 2.4 DNA extraction and Illumina MiSeq sequencing

For each sample, total DNA was extracted from 0.5 g soil using the PowerSoil^®^ DNA Isolation Kit (MoBio Laboratories Inc. Carlsbad, CA, USA) following the manufacturer’s instructions. The concentration and quality of DNA were measured by Nanodrop 2000 (Thermo Scientific, Wilmington, DE, USA). Before performing PCR amplification, the DNA sample was diluted to 10 ng/μL. The 16S rRNA V4–V5 regions were sequenced for bacterial communities with the primer pair 515F (5’-GTGCCAGCMGCCGCGGTAA-3’) and 909R (5’-CCCCGYCAATTCMTTTRAGT-3’). Fungal communities were assessed by using the ITS1 region of the rRNA operon with the primer pair ITS4 (5’- TCCTCCGCTTATTGATATGC-3’) and gITS7F (5’-GTGARTCATCGARTCTTTG-3’). Sequencing was conducted on an Illumina MiSeq2500 platform by Novogene (Beijing, China).

### 2.5 Statistical analysis

Statistical analyses were conducted using the R statistical software (R version 4.0.2, R Core Team, Vienna, Austria). Unless otherwise stated, statistical significance was set at *P* < 0.05. Differences in the plant above-ground biomass, litter biomass, soil properties, microbial α-diversity indices, and parameters of microbial co-occurrence networks were tested using one-way analyses of variance (ANOVA) followed by Tukey’s honestly significant difference (HSD) test. Normality and homogeneity of the distribution of residuals were verified and log-transformations performed when necessary. Linear regression analysis was used to evaluate the relationships between EMF and microbial variables. Principal coordinate analysis (PCoA) and permutational multivariate analysis of variance (PERMANOVA; based on Bray-Curtis distance) were used to determine significant differences in microbial communities at the various restoration modes. Metastat analysis was used to detect significant differences among treatments in term of bacteria and fungi at the phylum level. Redundancy analysis (RDA) was performed with a Monte Carlo permutation test (999 permutation) to identify soil properties that influence the microbial community structure. We performed random forest analysis to evaluate important predictors of ecosystem multifunctionality among bacterial Shannon and fungal Shannon diversity indices, bacterial richness (Chao1 index), fungal richness (Chao1 index), bacterial composition, and fungal composition. The composition of microbial communities was estimated based on Bray–Curtis distances between samples. All predictors and response variables were standardized before analyses using the Z-core to interpret parameter estimates on a comparable scale. Random forest analysis was performed using the “randomForest” package, with the significance of the model and each predictor evaluated using the “rfPermute” packages.

The co-occurrence network was constructed using the “microeco” package based on the spearman correlation matrix (Liu et al., 2021). Only the relative abundance of OTU > 0.01% was adopted in the analyses. The random matrix theory (RMT) was achieved to identify 0.79 and 0.80 as the thresholds for bacterial and fungal networks, respectively. Here, the *P* -value cutoff for statistical significance was set to 0.01. Microbial network modularity index of each sample was implemented using the subgraph function of the “microeco” package. Visualization of the microbial co-occurrence network was obtained using the interactive platform “Gephi”.

## 3 Results

### 3.1 Effects of restoration on ecosystem functioning

Long-term restoration had a positive effect on ecosystem functions (**Table 2**). Restoration modes had a significant effect on Plant AGB, SOM, TN, AN, DOC and DON, MBC and MBN, NAG, and ACP (*P* < 0.05), but had no significant effect on TP, AP, and CBH (*P* > 0.05). Treatment SG significantly increased litter biomass and BG on average by 484.9 and 585.3, respectively, relative to the CK (*P* < 0.05). Treatments MG and SA significantly decreased soil pH (*P* < 0.05). Compared with the CK treatment, treatments MG, SA, and SG significantly increased the multifunctionality index on average by 0.57, 0.23 and 0.76, respectively (*P* < 0.05).

**Table 2:** Effects of different restoration modes on soil basic properties and ecosystem functions.

Variables	CK	MG	SA	SG
AGB (g m^-2^)	9.4 ± 10.2 b	275.7 ± 63.0 a	32.6 ± 19.2 b	201.8 ± 39.6 a
Litter biomass (g m^-2^)	0.6 ± 0.4 b	54.3 ± 25.2 b	328.8 ± 218.2 ab	485.5 ± 239.4 a
pH	6.81 ± 0.08 a	6.33 ± 0.07 b	6.27 ± 0.18 b	6.68 ± 0.04 a
SOM (g kg^-1^)	3.53 ± 1.34 b	9.11 ± 1.64 a	5.84 ± 2.17 ab	9.29 ± 2.29 a
TN (g kg^-1^)	0.11 ± 0.02 b	0.35 ± 0.01 a	0.17 ± 0.02 b	0.39 ± 0.08 a
TP (g kg^-1^)	0.16 ± 0.00 a	0.17 ± 0.00 a	0.17 ± 0.01 a	0.18 ± 0.01 a
AN (mg kg^-1^)	10.32 ± 7.43 b	79.54 ± 9.19 a	29.39 ± 1.04 b	62.62 ± 15.07 a
AP (mg kg^-1^)	9.86 ± 1.87 a	8.85 ± 0.44 a	9.85 ± 2.52 a	11.64 ± 3.01 a
DOC (mg kg^-1^)	51.9 ± 0.9 c	82.7 ± 4.9 b	67.3 ± 3.5 bc	106.4 ± 15.4 a
DTN (mg kg^-1^)	14.9 ± 0.8 b	24.9 ± 2.5 a	16.3 ± 1.2 b	32.2 ± 6.8 a
MBC (mg kg^-1^)	171.1 ± 36.7 b	237.0 ± 10.2 a	205.0 ± 18.8 ab	238.3 ± 22.0 a
MBN (mg kg^-1^)	23.8 ± 4.2 b	51.4 ± 6.3 a	30.8 ± 5.3 b	48.3 ± 7.6 a
BG (nmol g^−1^ h^−1^)	196.8 ± 56.4 b	381.6 ± 73.8 b	365.1 ± 86.6 b	682.1 ± 198.8 a
CBH (nmol g^−1^ h^−1^)	63.5 ± 18.6 a	88.8 ± 30.7 a	97.5 ± 47.8 a	100.3 ± 11.8 a
NAG (nmol g^−1^ h^−1^)	74.6 ± 38.9 b	209.5 ± 44.1 a	121.3 ± 50.2 ab	232.2 ± 80.7 a
LAP (nmol g^−1^ h^−1^)	31.2 ± 13.1 a	34.7 ± 6.1 a	28.4 ± 18.2 a	31.7 ± 7.4 a
ACP (nmol g^−1^ h^−1^)	96.9 ± 25.4 b	663.0 ± 155.1 a	250.9 ± 26.4 b	722.8 ± 119.8 a
Multifunctionality (Zcore)	0.004 ± 0.003 c	0.578 ± 0.121 a	0.233 ± 0.041 b	0.768 ± 0.152 a

Values are mean ± standard deviation (n = 4). Values followed by different letters in superscript are significantly different among treatments (P < 0.05). CK: extremely degraded grassland. MG: planting mixed grasses. SA: planting shrub with Salix cupularis alone (SA). SG: planting shrub with Salix cupularis plus mixed grasses. AGB, above-ground biomass; SOM, soil organic matter; TN, total nitrogen; TP, total phosphorus; AN, available nitrogen; AP, available phosphorus; DOC, dissolved organic carbon; DTN, dissolved total nitrogen; MBC, microbial biomass carbon; MBN, microbial biomass nitrogen; BG, β-1,4-glucosidase; CBH, β-D-cellobiosidase; NAG, β-1,4-N-Acetyl-glucosaminidase; LAP, leucine aminopeptidase; ACP, acid phosphatase.

### 3.2 Effects of restoration on the diversity and composition of microbial communities

**Figure 1:**
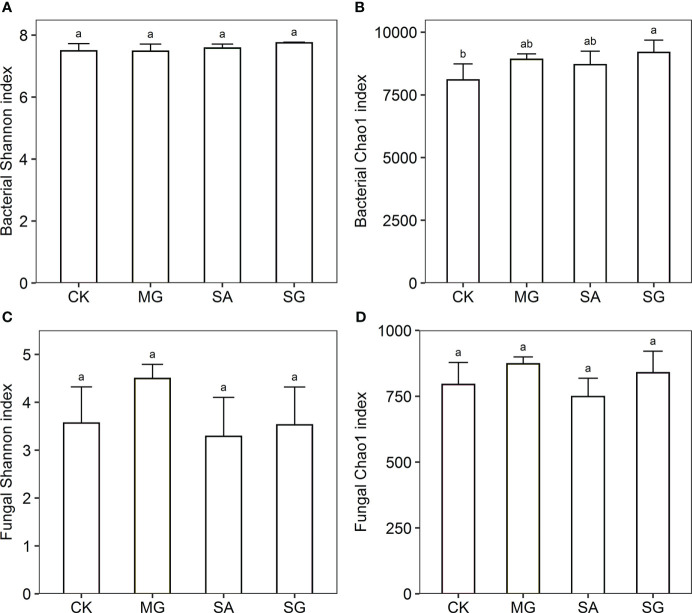
Responses of alpha diversity of bacterial and fungal communities to different restoration modes. **(A)** bacterial Shannon index, **(B)** bacterial Chao1 index, **(C)** fungal Shannon index, **(D)** fungal Chao1 index. CK: extremely degraded grassland. MG: planting mixed grasses. SA: planting shrub with *Salix cupularis* alone (SA). SG: planting shrub with *Salix cupularis* plus mixed grasses. Different lowercase letters indicate significant differences among treatments (*P* < 0.05).

The Shannon index of bacteria was higher in the SG treatment than in the CK, MG, and SA treatments. The Chao1 index of bacteria was significantly higher in the SG than the CK treatment (*P* < 0.05) (**Figure 1**). The Chao1 index of fungi in the MG and SG treatments were higher than in the CK treatment; SA was lower than in CK; and there were no significant differences between MG, SA and SG.

**Figure 2:**
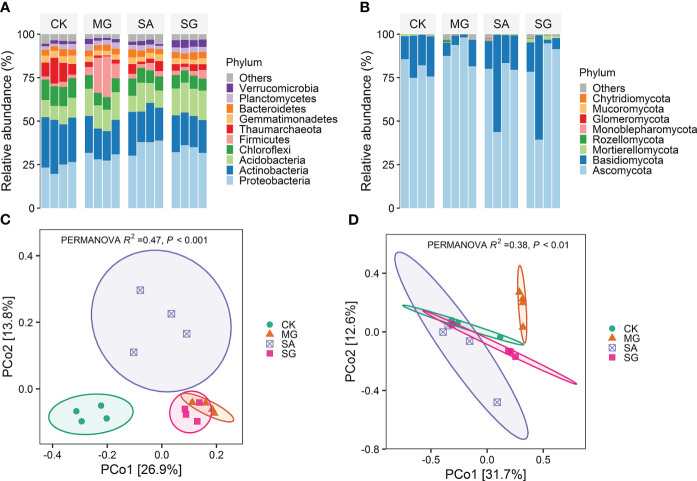
Effects of different restoration modes on soil bacterial and fungal communities. **(A)** Distribution of dominant bacterial groups at the phylum level. **(B)** Distribution of dominant fungal groups at the phylum level. **(C)** Principial coordinates analysis (PCoA) of bacterial community composition based on Bray-Curtis distances. **(D)** Principial coordinates analysis (PCoA) of fungal community composition based on Bray-Curtis distances. CK: extremely degraded grassland. MG: planting mixed grasses. SA: planting shrub with *Salix cupularis* alone (SA). SG: planting shrub with *Salix cupularis* plus mixed grasses.

Soil bacterial communities under different restoration modes were primarily comprised of members of the phyla Proteobacteria, Actinobacteria, Acidobacteria, Chloroflexi, and Firmicutes (**Figure 2A**). Treatments MG, SA and SG significantly decreased the relative abundance of Chloroflexi on average by 3.8%, 2.9% and 4.1% relative to the CK treatment, respectively (*P* < 0.05).Compared with the CK, the MG significantly increased the relative abundance of Acidobacteria by 4.36%, but significantly decreased the relative abundance of Actinobacteria, Gemmatimonadetes and Thaumarchaeota by 8.2%, 1.2%, and 8.2%, respectively (*P* < 0.05). The SG significantly increased the relative abundance of Proteobacteria, Acidobacteria, and Verrucomicrobia by 10.2%, 6.4%, and 2.4%, respectively (*P* < 0.05). Ascomycota (~79%) and Basidiomycota (~19%) were the major fungal phyla (**Figure 2B**). At the phylum level, the fungal community compositions remained relatively stable among different treatments.

The PCoA analyses demonstrated restoration altered the overall patterns of both bacterial and fungal communities (**Figures 2C, D**). The PERMANOVA test showed significant differences between treatments. For bacteria, samples from MG, SA and SG clustered separately from CK samples. For fungi, MG samples clustered separately from CK ones. RDA analyses indicated the the first two components explained 50.9% and 50.7% of the total variability for bacterial and fungal community structure, respectively (**Supplementary Figure S2**). Soil pH, SOM, TN and DTN were the important soil properties controlling the bacterial community structure (*P* < 0.05). Moreover, SOM contents significantly correlated with the fungal communities (*P* < 0.01) (**Supplementary Table S2**).

### 3.3 Contribution of the diversity and composition of microbes to EMF

**Figure 3:**
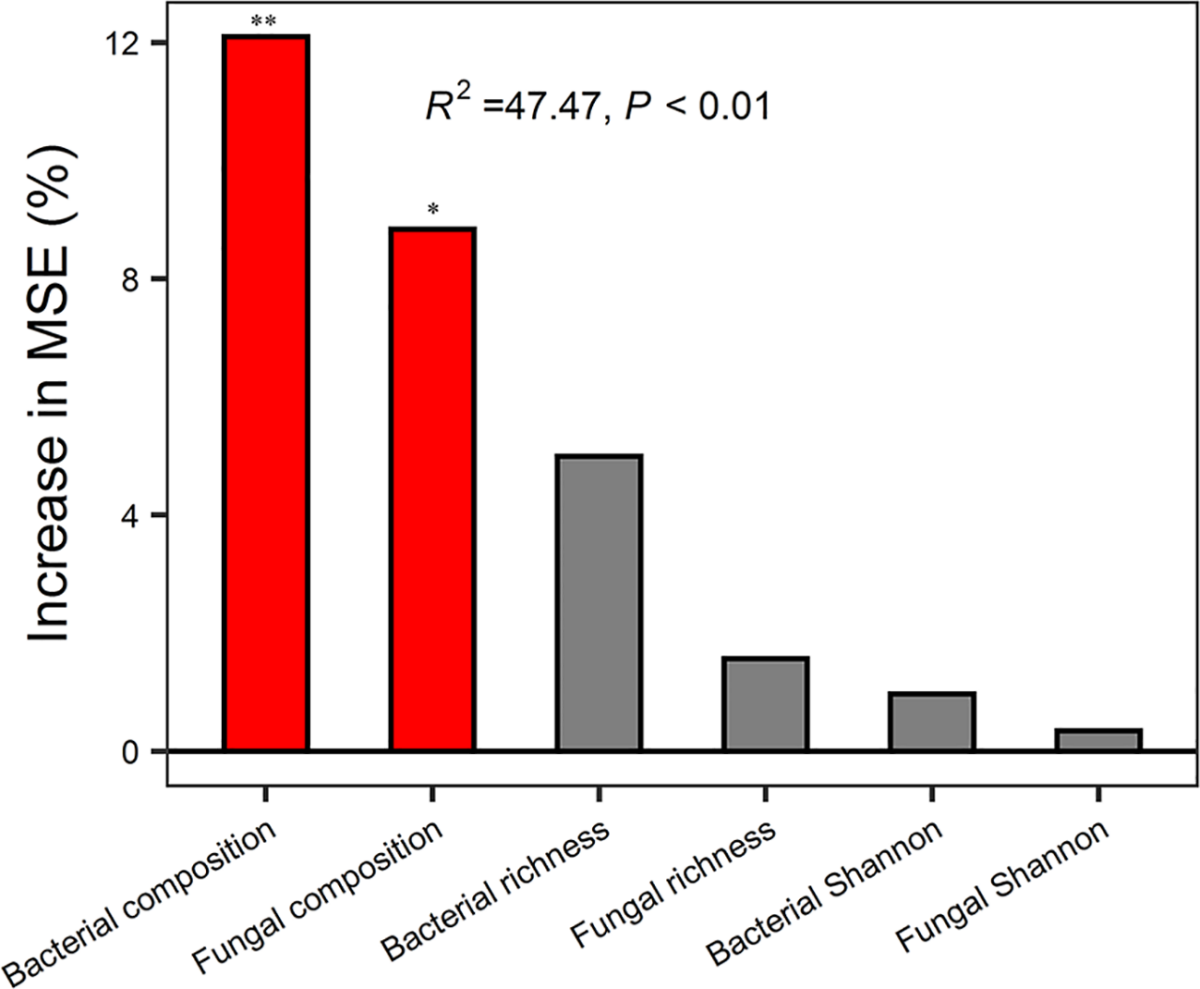
Random Forest regression model showing the mean predictor importance (% of increase of MSE) of microbial drivers of ecosystem multifunctionality. MSE, represents the mean square error. ^*^ indicates *P* < 0.05 and ^**^ indicates *P*< 0.01 (both meaning that the associated driver had a significant effect on multifunctionality). Bacterial composition and fungal composition were represented by scaling 1, the first component of PCoA analysis. Bacterial richness and fungal richness were represented by Chao1 index.

**Figure 4:**
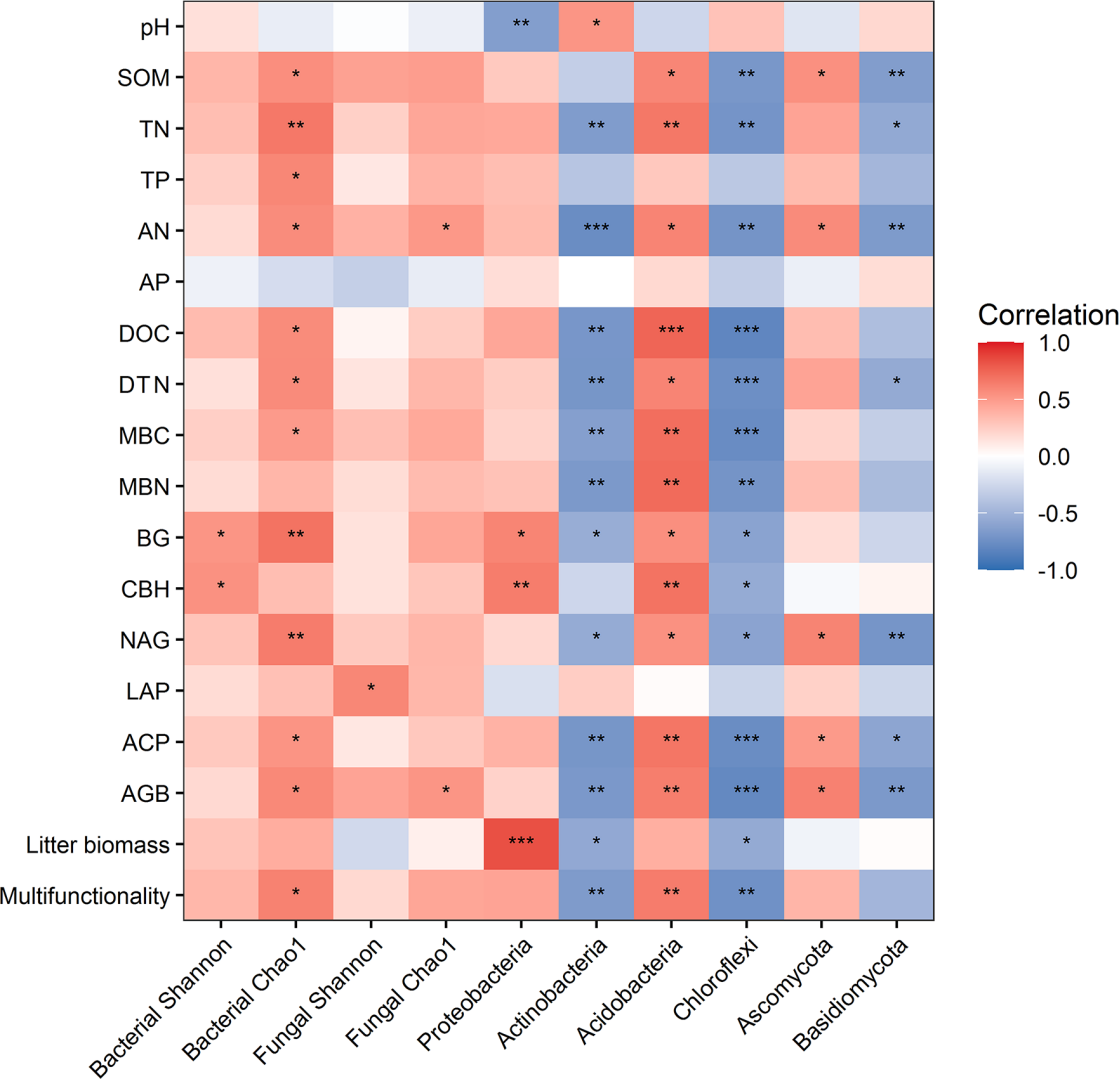
Relationships between ecosystem functions and the diversity and dominant phyla of microbial communities. ^*^ indicates *P* < 0.05, ^**^ indicates *P*< 0.01, ^***^ indicates *P* < 0.001.

Random forest analysis indicated that bacterial composition and fungal composition controlled EMF rather than the richness and diversity of bacteria and fungi (**Figure 3**). The EMF index was significantly positively associated with the Chao1 index of bacteria (*P* < 0.05). Plant above-ground biomass and AN were positively associated with the Chao1 index of bacteria and fungi (*P* < 0.05). Acidobacteria was positively correlated with multifunctionality index, SOM, TN, AN, DOC, DTN, MBC, MBN, BG, CBH, NAG, ACP, and AGB (*P* < 0.05). Proteobacteria was significantly positively correlated with BG, CBH and litter biomass, but negatively correlated with pH (*P* < 0.05). Ascomycota was positively correlated with SOM, AN, NAG, ACP and AGB (*P* < 0.05) (**Figure 4**).

### 3.4 Effects of restoration on microbial co-occurrence network

**Figure 5:**
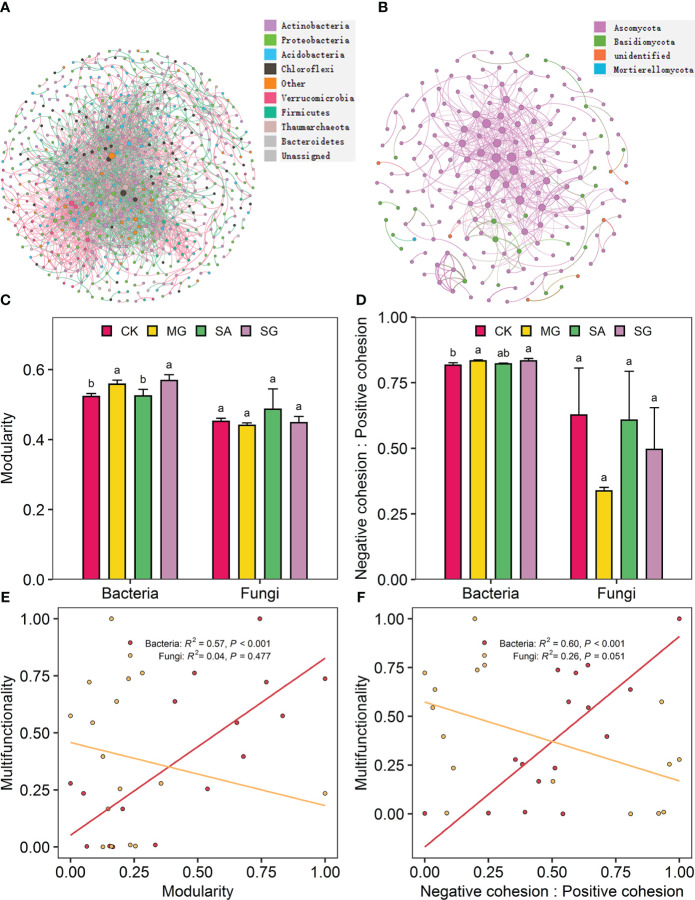
Co-occurrence patterns in soil bacterial and fungal communities as affected by restoration and the relationships of ecosystem multifunctionality and the stability of bacterial and fungal communities. Co-occurrence network of soil bacterial communities **(A)** and fungal communities **(B)**. The modularity **(C)** and the ratio of negative cohesion to positive cohesion **(D)** of soil bacteria and fungi co-occurrence patterns from extremely degraded grassland (CK), planting mixed grasses (MG), planting shrub with *Salix cupularis* alone (SA), and planting shrub with *Salix cupularis* plus mixed grasses (SG). The relationships of ecosystem multifunctionality to modularity **(E)** and the ratio of negative cohesion to positive cohesion **(F)** of soil bacteria and fungi co-occurrence patterns.

The co-occurrence network of bacterial and fungal communities is shown in **Figures 5A, B**. Compared with the CK treatment, the MG, SA, and SG treatments increased the modularity of bacterial network. This indicates that bacterial communities in restoration treatments were more compartmentalized than in degraded treatments (**Figure 5C**). Treatments MG, SA, and SG increased the ratio of negative to positive cohesion of bacterial network relative to the CK (**Figure 5D**). Restoration modes had no significant effect on the fungal network stability (*P* > 0.05). Correlation analysis showed that the EMF index was positively correlated with the modularity (R^2 ^= 0.57, *P* < 0.001) and the ratio of negative cohesion to positive cohesion (R^2 ^= 0.60, *P* < 0.001) of bacteria network (**Figures 5E, F**). The modularity and the ratio of negative cohesion to positive cohesion of bacterial network were positively correlated with SOC, TN, AN, DOC and DTN (*P* < 0.05) (**Supplementary Figure S3**).

## 4 Discussion

### 4.1 Responses of ecosystem functions to ecological restoration

In alpine degraded grasslands, the major challenge is to develop the sustainable and effective restoration mode to solve a series of ecosystem degradation problems caused by human disturbances and climate change (Xu Y et al., 2021). Our findings demonstrate that long-term restoration have a positive effect on multifunctionality index and most individual ecosystem functions, including plant biomass, soil fertility, and microbial activities. These results align with previous studies (Xiao et al., 2021; Zhou et al., 2022), which also indicated that different restoration modes exert varying effects on ecosystem multifunctionality.

Specifically, treatments MG and SG exhibited a significantly higher positive effect on the multifunctionality index compared to the CK and SA treatments. This finding supports the argument made by Zhao et al. (2022) that mixed-species restoration is a viable strategy for restoring ecosystem functions and services in degraded ecosystems. Furthermore, in terms of each component of ecosystem multifunctionality, MG and SG treatments were more effective than the SA treatment in restoring AGB, SOM, TN, enzymes activities and MBC. Several factors can account for these differences. Firstly, compared to the SA treatment, the higher vegetation coverage in MG and SG treatments facilitates the accumulation, distribution, and cycling of soil nutrients, consequently promoting plant growth (Zhao et al., 2022). Secondly, the increase vegetation coverage in MG and SG treatments mitigates wind or rainfall erosion, enhancing soil stability and improving soil quality, thereby stimulating the growth of vegetation and microbes (Singh et al., 2018; Dong et al., 2022). Thirdly, the presence of different growth forms and co-dominant species in MG and SG treatments may lead to higher amounts of root exudates, also improving soil properties and microbial activities (Chen et al., 2019; Huang et al., 2022). Additionally, treatments SA and SG exhibited increased litter biomass, as shrubs contribute significantly to the input of fallen leaves and twigs.

### 4.2 Responses of soil microbial communities to ecological restoration

The alpha diversity of bacteria and fungi in the MG and SG treatments was higher than in the SA and CK treatments. One possible explanation is that the higher soil fertility of MG and SG created a more suitable microenvironment for bacteria and fungi to survive, and consequently enhanced the diversity of soil bacterial and fungal community (Lu et al., 2022). Moreover, mixtures of plant species in the SG and MG treatments likely provided the microbes with greater accessibility to organic materials and a variety of root exudates, which results in more niches to support higher diversity of bacterial and fungal taxa (Zhao et al., 2022).

Soil properties are the key factors influencing the structure of soil microbial communities (Bahram et al., 2018). Our results showed that soil pH was an important factor altering soil bacterial communities under varying conditions of ecological restoration, also consistent with studies conducted at either local or larger spatial scales (Bahram et al., 2018; Delgado-Baquerizo et al., 2018; Lu et al., 2022). This presumably due to the relatively narrow optimal pH for bacterial growth (Shu et al., 2022). SOM, TN and DTN were the other major factors influencing the bacterial community structure. SOM was also a key factor influencing the fungal community structure. Our results are in line with those of Lu et al. (2022), who found that soil microbial community structure was closely related to soil nutrients and particularly SOM content.

### 4.3 Relationships between EMF and the diversity and composition of soil microbial communities

Clarifying how microbial diversity and composition influence ecosystem multifunctionality in ecological restoration is critical for restoring and managing degraded grassland ecosystems. We observed positive relationships between microbial diversity with ecosystem multifunctionality, as well as with most individual functions, which is consistent with previous studies (Chen L et al., 2021; Jing et al., 2015). Higher diversity can promote better performance in ecosystem functions due to the potential high functional redundancy of microbial communities (Le Bagousse-Pinguet et al., 2019). We also found positive relationships between soil microbial diversity and EMF, although not all soil microorganisms played an equally important in regulating EMF. Our findings suggest that the diversity and composition of bacterial communities, rather than fungal communities, drove EMF. Soil bacteria are considered the main component of microbial communities, accounting for 99% of total soil microorganisms in degraded grassland ecosystems (Zhang et al., 2021). Soil bacteria encompass a relatively broad taxonomic grouping with diverse traits and functions; while fungi represent a narrower taxonomic grouping with a more limited range of functions (Jing et al., 2015; Mori et al., 2016). Additionally, we observed a significant positive correlation between plant above-ground biomass and the Chao1 index of bacteria and fungi, confirming that the importance of soil bacterial and fungal richness in biomass production (Yang et al., 2021).

The composition of bacterial and fungal communities emerged asa strong predictor of ecosystem multifunctionality. In this study, the phyla Proteobacteria, Actinobacteria, Acidobacteria, and Chloroflexi were the most abundant bacterial taxa, which is consistent with findings from local and regional studies (Baubin et al., 2019; Feng et al., 2020). The higher relative abundance of Proteobacteria and Acidobacteria may have contributed to the recovery of ecosystem functions. These bacterial groups are associated with soil health and quality due to their effects on various biogeochemical processes and strongly influenced by degradation and land use (Crowther et al., 2019). Proteobacteria tend to thrive in nutrient-enriched environments and play a critical role in complex and labile C decomposition (Yao et al., 2017). Acidobacteria is a keystone taxa in soil, involved in soil organic matter decomposition (Costa et al., 2020), nitrogen cycling, and plant growth promotion (Eichorst et al., 2018; Kalam et al., 2020). Actinobacteria and Chloroflexi are defined as oligotrophs, thriving in soils with low nutrients (Chen L.F et al., 2021; Fu et al., 2022). In contrast to degradation, ecological restoration can improve soil organic matter content and nutrient availability, which may explain the decline in the relative abundance of Actinobacteria and Chloroflexi. Furthermore, Ascomycota showed a significant positive correlation with SOM, NAG, ACP and plant above-ground biomass, supporting its critical role in nutrient cycling and supporting plant growth (Challacombe et al., 2019).

Several studies have concluded that temperature, precipitation, inorganic nutrients, and organic matter exhibit marked seasonal differences, which strongly affects ecosystem properties (Singh et al., 2018; Lin et al., 2021; Xu C et al., 2021). Alsterberg et al. (2017) observed that both ecosystem multifunctionality and the structure and diversity of soil bacterial communities are significantly affected by seasonal dynamics. Du et al. (2019) found clear seasonal changes in tthe biomass and necromass of fine roots during vegetation succession. Yang et al. (2022) demonstrated that plant litter inputs, soil carbon availability, microbial functional genes, and the composition and diversity of soil microbial communities exhibit strongly seasonal varations. Moreover, the relationships between soil microbial composition and soil-related functions are dependent on the sampling season. In this study, we focused solely on investigating the effects of different long-term restoration modes on ecosystem functions and microbial communities during the summer season. Due to the lack of observational data, we cannot determine how restoration impacts ecosystem functions and its relationship with the composition and diversity of bacterial and fungal communities in different seasons. Therefore, future studies should consider seasonal variability to provide a more comprehensive evaluation.

### 4.4 Linkages between EMF and soil microbial network stability under ecological restoration

The study of microbial stability is fundamental but has been largely overlooked in ecological investigations of restored ecosystems. Exploring how long-term ecological restoration may influence microbial co-occurrence networks provides new insights into understanding the stability of microbial communities. Community stability can be characterized by various network topological properties, such as modularity and cohesion (Hernandez et al., 2021). Modularity quantifies how strongly taxa are compartmentalized into groups of interacting/co-occurring taxa. Generally, communities with higher network modularity tend to be more stable as they have more functionally interrelated members (Ye et al., 2021). Meanwhile, the ratio of negative to positive cohesion among microbial taxa is a good indicator of community stability, as stability is often associated with negative interactions. A high proportion of negative correlations within a community is considered to be more stable (Coyte et al., 2015). In this study, we observed that the ratio of negative to positive cohesion of bacterial network increased in MG, SA, and SG, suggesting that negative associations between taxa dominate in these restoration treatments. Additionally, the modularity of bacterial network were significantly higher in the MG and SG treatments compared to the CK and SA treatments, indicating that MG and SG can stabilize bacterial networks. The increase stability of bacterial network in MG and SG treatments may result from enhanced resource availability, which promotes microbial richness and stability (**Supplementary Figure S3**). This finding is consistent with recent research indicating that microbes in low-stress habitats exhabit high modularity and higher ratios of negative to positive cohesion compared to communities in high-stress habitats (Hernandez et al., 2021). Importantly, we found a significant positive correlation between EMF and the modularity and the ratio of negative cohesion to positive cohesion of bacteria. This suggests that ecological restoration could stabilize bacterial communities and improve their ecosystem functions by promoting the compartmentalization of bacterial associations and fostering bacterial communities dominated by negative associations.

It is crucial to recognize that the effects of ecological restoration on the structure, functions and stability of degraded ecosystems occurs over time, so the evaluation of the influence and effectiveness must consider the necessary time frame (Hastings, 2016). Previous studies indicate that the positive effects of ecological restoration on soil fertility and biodiversity can increase with restoration time. For instance, Guo et al. (2021) found that soil multifunctionality, plant richness, and microbial richness increased concomitantly with restoration time. Our previous research based on a 34-year time series found that soil fertility and plant biomass increased with the increasing plantation period of shrub with *Salix cupularis* alone (Hu et al., 2018). Huang et al. (2019) conducted a meta-analysis and revealed that the biodiversity of the restored state increased by 34%, 51%, and 122% for restoration durations of 0-10, 10-20 and >20 years, respectively, compared to the unrestored (degraded) state. However, evidence suggests that biodiversity does not necessarily increase with restoration time. For instance, van der Heyde et al. (2020) found that both soil bacterial diversity and fungal diversity did not consistently increase with restoration age at different sites. Additionally, a recent global meta-analysis showed that the positive effects of restoration on plant biomass, soil carbon, soil fertility, and SOM decomposition increased with increasing of restoration age, while plant diversity and microbial biodiversity did not (Zhou et al., 2022). The community structure and diversity of soil microorganisms can vary significantly on the scale of days, months, and years, making it difficult to identify a single general trend (Chernov and Zhelezova, 2020). In this study, we observed that MG and SG treatments increased microbial richness, stabilize bacterial network, and promote multiple ecosystem functions after 14-years establishment period. However, it remains unclear whether the positive effect of these modes on microbial richness and network stability will presist across different restoration ages. Considering the significance of microbial community richness and network stability for ecosystem stability (Yang et al., 2018; Yuan et al., 2021), we recommend that future studies investigate the effects of different ecological restoration modes on microbial richness and microbial network stability over a longer time span to gain a more comprehensive understanding.

## 5 Conclusions

Our study revealed that three restoration modes had a significant positive effect on ecosystem multifunctionality, compared to degraded (unrestored) grassland. We observed that the composition and diversity of bacterial communities played a crucial role in determining ecosystem multifunctionality, while the influence of fungal communities was comparatively less pronounced. Furthermore, our results highlight the strong association between soil bacterial network stability and ecosystem multifunctionality. Notably, the combination of planting shrubs and grasses demonstrated to be an effective restoration mode for enhancing ecosystem multifunctionality, as well as promoting microbial diversity and stability. These findings provide valuable insights into the role of soil microbiota in driving ecosystem multifunctionality within the context of ecological restoration. Moreover, they have significant implications for the implementation of large-scale restoration projects and strategies aimed at enhancing multifunctionality in ecosystems.

## Data availability statement

The original contributions presented in the study are publicly available. This data can be found here: https://www.ncbi.nlm.nih.gov/bioproject/PRJNA945234.

## Author contributions

XS and WL: Writing - original draft, Funding acquisition, Writing – review &editing, Visualization, Methodology. YH: Funding acquisition, Writing - review & editing. LX, YYZ, KF, YLZ and WZ: Writing - review & editing. All authors contributed to the article and approved the submitted version.
