# Supplementary material for: Ecosystem multifunctionality and soil microbial communities in response to ecological restoration in an alpine degraded grassland
Source: Front Plant Sci. 2023 Aug 1;14:1173962. doi: 10.3389/fpls.2023.1173962 (PMC10431941; doi:10.3389/fpls.2023.1173962)
Supplement: Supplementary file 1 [file DataSheet_1.docx]

**Supplementary materials**

**
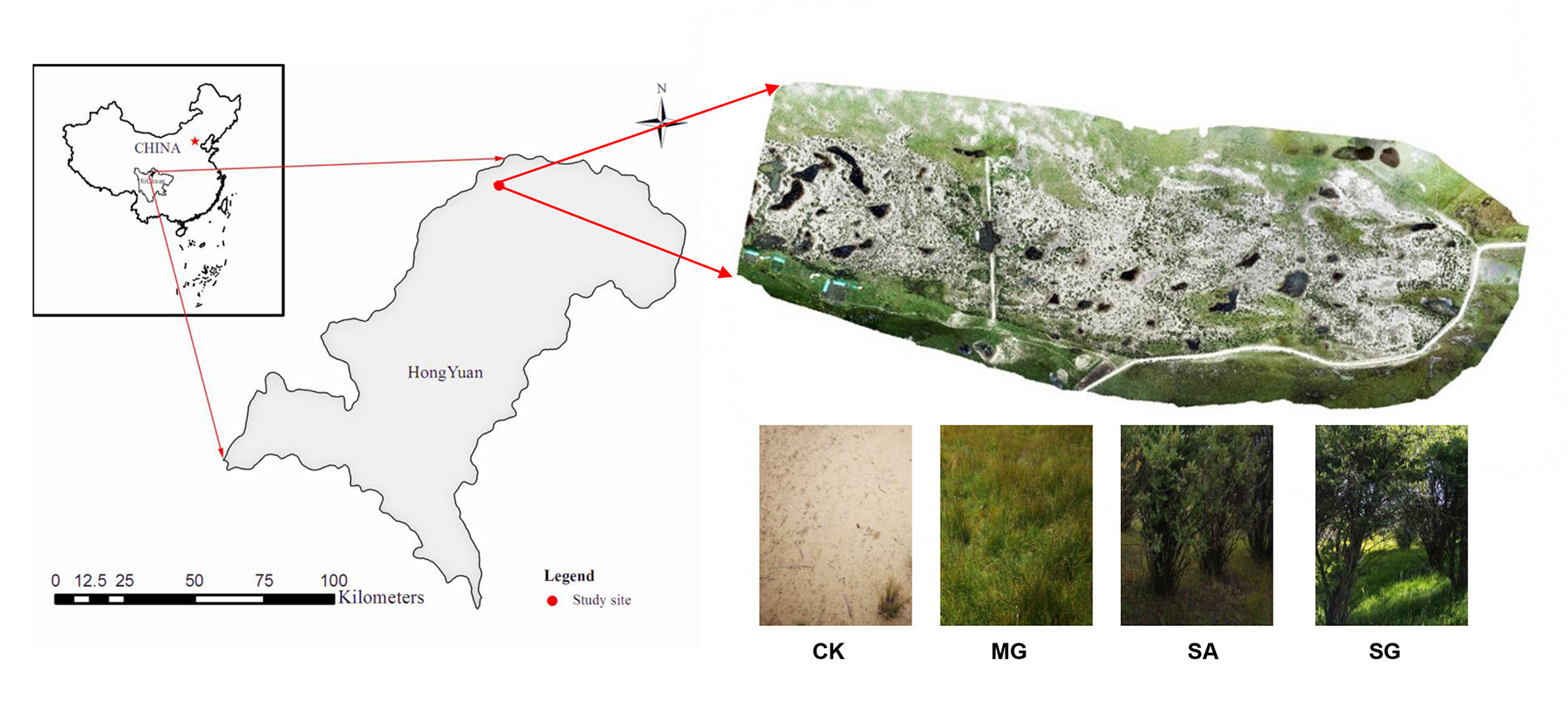
**

**Supplementary Figure S1** Location of the study area and image of ecological restoration modes. Note: CK, extremely degraded grassland; MG, planting mixed grasses; SA, planting shrub with *Salix cupularis* alone; SG, planting shrub with *Salix cupularis* plus grasses.


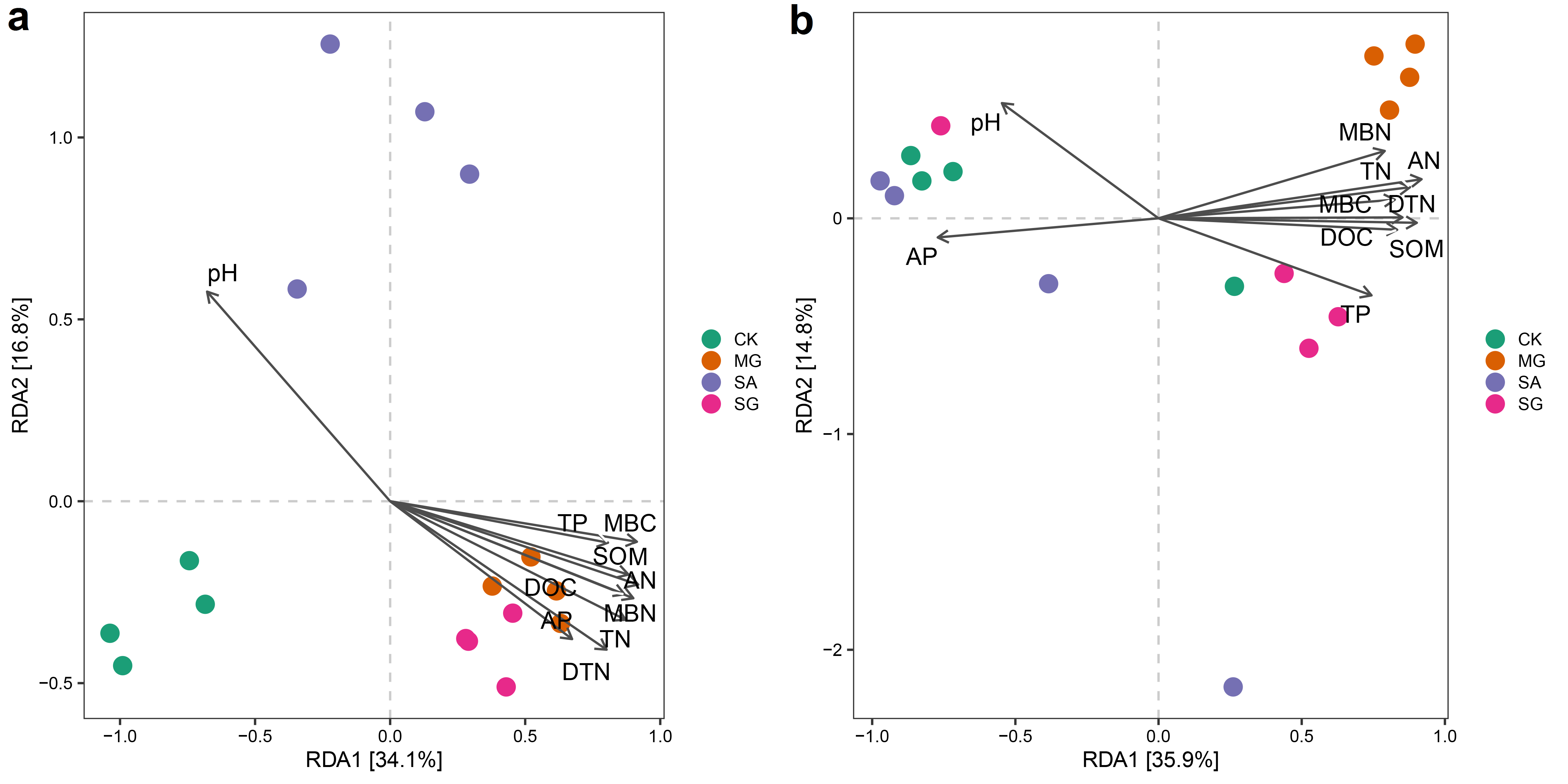


**Supplementary Figure S2** Redundancy analysis (RDA) of bacterial (**a**) and fungal (**b**) community changes with soil properties. CK: extremely degraded grassland. MG: planting mixed grasses. SA: planting shrub with *Salix cupularis* alone (SA). SG: planting shrub with *Salix cupularis* plus mixed grasses. SOM: soil organic matter; TN: total nitrogen; TP: total phosphorus; AN: available nitrogen; AP: available phosphorus; DOC: dissolved organic carbon; DTN: dissolved total nitrogen; MBC: microbial biomass carbon; MBN: microbial biomass nitrogen.
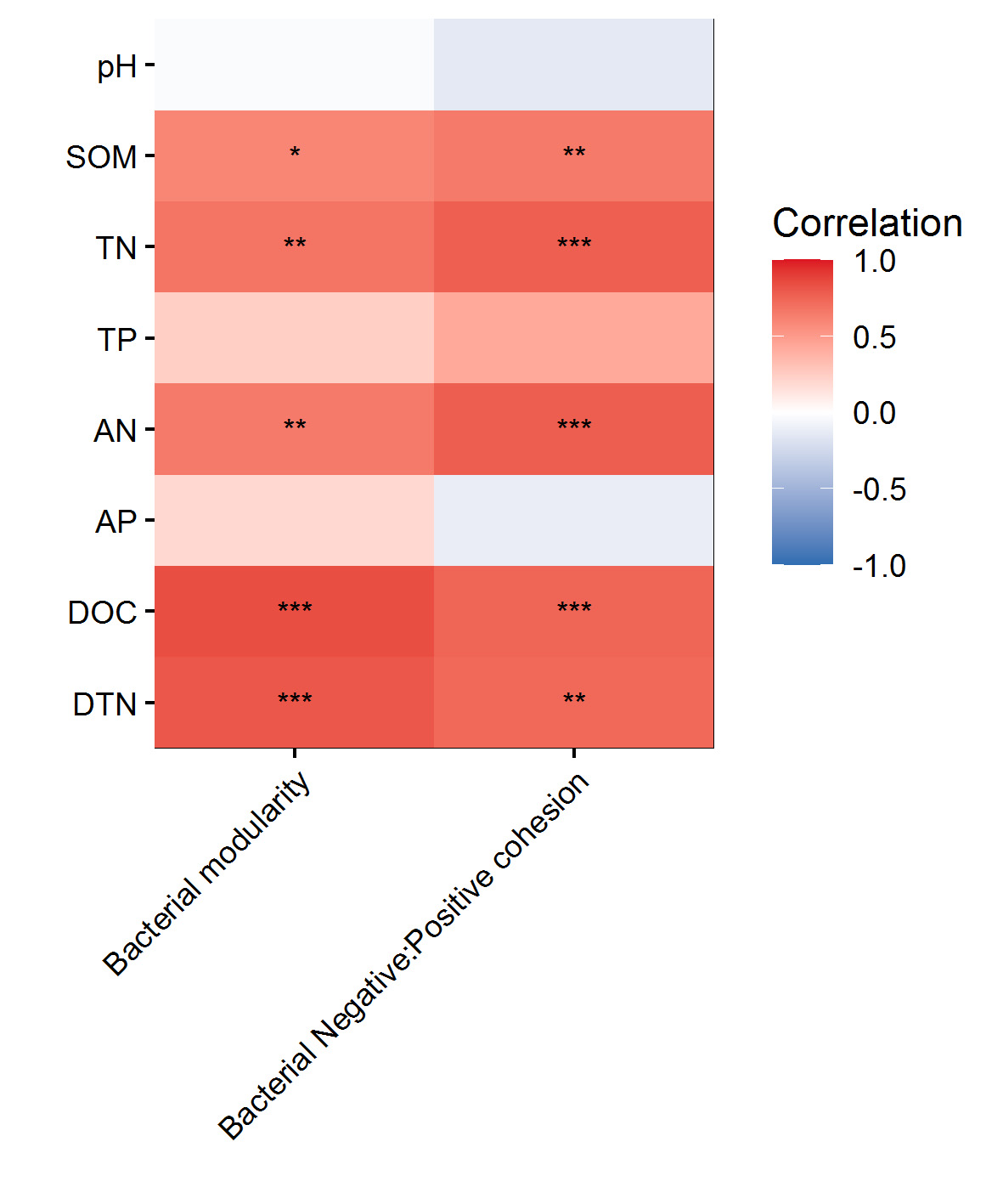


**Supplementary Figure S3** The relationships of soil properties to modularity and the ratio of negative cohesion to positive cohesion of soil bacteria co-occurrence patterns. SOM: soil organic matter; TN: total nitrogen; TP: total phosphorus; AN: available nitrogen; AP: available phosphorus; DOC: dissolved organic carbon; DTN: dissolved total nitrogen

**Supplementary materials**

**Supplementary Table S1** A detailed description of soil extracellular enzymes included in this study.

| Enzyme | EC | Function | Abbreviation |
| --- | --- | --- | --- |
| β-1,4-glucosidas | 3.2.1.21 | Hydrolysis of cellulose | BG |
| β-D-cellobiosidase | 3.2.1.91 | Hydrolysis of cellulose | CBH |
| β-1,4-N-Acetyl-glucosaminidase | 3.2.1.30 | Hydrolysis of chitooligosaccharides | NAG |
| Leucine amino peptidase | 3.4.11.1 | Cleaving of peptide bonds in proteins | LAP |
| Acid phosphatase | 3.1.3.2 | Cleaving of PO_4_^3-^ from P-containing organic matter | ACP |

The activities of β-glucosidase, β-D-cellubiosidase, and N-acetyl-β-glucosaminidase were measured using p-nitrophenyl-β-D-glucopyranoside, 4-nitrophenyl-β-D-cellobioside, and N-acetyl-D-glucosamine as a substrate, respectively. After incubation at 37 °C for 1 h, the amount of p-nitrophenol (PNP) was measured at 400 nm. Soil leucine amino peptidase was measured using L-Leucine-p-nitroanilide as a substrate. After incubation at 30 °C for 24 h, the amount of p-Nitroaniline was measured at 405 nm. Soil acid phosphatase was measured using disodium phenyl phosphate as a substrate. After incubation at 37 °C for 24 h, the amount of phenol was measured at 570 nm.

**Supplementary Table S2** Correlations between bacterial and fungal community composition and soil properties.

|  | Bacteria | | Fungi | |
| --- | --- | --- | --- | --- |
|  | F | Pr(>F) | F | Pr(>F) |
| pH | 2.64923 | **0.001** | 1.0855 | 0.327 |
| SOM | 2.6357 | **0.002** | 3.6247 | **0.002** |
| TN | 1.6385 | **0.035** | 1.6334 | 0.087 |
| TP | 1.1369 | 0.242 | 1.6969 | 0.056 |
| AN | 1.2452 | 0.167 | 1.1522 | 0.294 |
| AP | 1.058 | 0.364 | 1.4285 | 0.144 |
| DOC | 1.2157 | 0.154 | 1.5223 | 0.122 |
| DTN | 1.604 | **0.034** | 1.5783 | 0.079 |
| MBC | 1.1818 | 0.198 | 0.949 | 0.486 |
| MBN | 1.0356 | 0.381 | 1.1712 | 0.265 |
